# Inexpensive Graphene Oxide Heaters Lithographed by Laser

**DOI:** 10.3390/nano9091184

**Published:** 2019-08-21

**Authors:** Francisco J. Romero, Almudena Rivadeneyra, Inmaculada Ortiz-Gomez, Alfonso Salinas, Andrés Godoy, Diego P. Morales, Noel Rodriguez

**Affiliations:** 1 Pervasive Electronics Advanced Research Laboratory, University of Granada, 18071 Granada, Spain; 2 Department of Electronics and Computer Technology, University of Granada, 18071 Granada, Spain; 3 Department of Analytical Chemistry, University of Granada, 18071 Granada, Spain; 4 Biochemistry and Electronics as Sensing Technologies Group, University of Granada, 18071 Granada, Spain

**Keywords:** flexible electronics, graphene oxide, heater, laser-scribing, thermal response

## Abstract

In this paper, we present a simple and inexpensive method for the fabrication of high-performance graphene-based heaters on different large-scale substrates through the laser photothermal reduction of graphene oxide (laser-reduced graphene-oxide, LrGO). This method allows an efficient and localized high level of reduction and therefore a good electrical conductivity of the treated films. The performance of the heaters is studied in terms of steady-state temperature, power consumption, and time response for different substrates and sizes. The results show that the LrGO heaters can achieve stable steady-state temperatures higher than 200 °C when a voltage of 15 V is applied, featuring a time constant of around 4 s and a heat transfer coefficient of ~200 °C cm^2^/W. These characteristics are compared with other technologies in this field, demonstrating that the fabrication approach described in this work is competitive and promising to fabricate large-scale flexible heaters with a very fast response and high steady-state temperatures in a cost-effective way. This technology can be easily combined with other fabrication methods, such as screen printing or spray-deposition, for the manufacturing of complete sensing systems where the temperature control is required to adjust functionalities or to tune sensitivity or selectivity.

## 1. Introduction

In recent years, heaters have attracted a growing interest because of the emergence of a broad spectrum of newly integrated sensing technologies, such as gas sensors or biosensors [1,2,3], which require a certain constant temperature or a programmable sequence of temperatures to operate or to achieve a proper performance [4,5]. In this regard, heaters are expected to play a notable role in the ubiquitous sensing of environmental and biological parameters. However, the major challenges facing their widespread use are related to the demanding requirements of this kind of application since, apart from the response, the recovery time, and the stability properties, these applications also require flexibility, lightness, low cost, transparency, or biocompatibility [6,7]. 

In this context, graphene and graphene related materials have attracted the attention of many researchers as an alternative to the conventional expensive indium tin oxide (ITO) heaters due to their outstanding electrochemical, mechanical, and optical properties [8]. Thus, different approaches for the fabrication of graphene-based heaters can be found in the literature. One of the most commonly used is based on the bottom-up production of graphene by the chemical vapor deposition (CVD) process, as presented by Kang et al. [9]. However, this approach suffers from large sheet resistance and therefore requires chemical doping and multiple transfer processes to be applied in large-scale manufacturing. To tackle this issue, other authors such as Lin et al. and Kang et al. [10,11] make use of hybrid structures of silver particles mixed with graphene, reducing the sheet resistance and increasing both steady-state temperature and response time. However, although some of these approaches report an adequate performance, the drawbacks of a complex fabrications process, slow thermal response, or high-power consumption limit their integration in end-user applications. For these reasons, a cost-effective and scalable process for the manufacturing of graphene-based heaters is still being sought.

In this work, we present a methodology for the fabrication of high-performance laser-reduced graphene-oxide (LrGO) heaters, which can be applied on different substrates (flexible or not). The laser reduction of GO [12,13], aiming to increase its conductivity, offers several advantages over other reduction processes, such as chemical or thermal ones [14]: (i) it does not require chemicals reagents to produce the reduction, making it an environmentally friendly technique, (ii) it allows the lithography of high-resolution patterns of reduced graphene oxide without the need for masks [15], therefore offering a simpler and more economical process, (iii) it also allows a surface-localized treatment of the GO without affecting the substrate, which increases its versatility, extending the range of suitable supporting materials [16]. 

SEM, Raman spectroscopy, as well as X-ray photoelectron spectroscopy (XPS) were used to study the quality of the synthesized material, and the electrothermal experiments show promising results in terms of power consumption, response time, and steady-state temperature when compared with other existing technologies. Thus, this fabrication approach paves the way to an extremely simple, inexpensive, and eco-friendly fabrication of graphene-based heaters. The paper is structured as follows. The fabrication of the LrGO heaters is described in Section 2 along with the materials and the characterization setups used. Section 3 presents structural, electrical, and thermal characterization of the LrGO heaters, comparing their key features with other approaches in the literature. Finally, the main conclusions are outlined in Section 4. 

## 2. Materials and Methods 

### 2.1. Fabrication of the rGO Films

**Figure 1:**
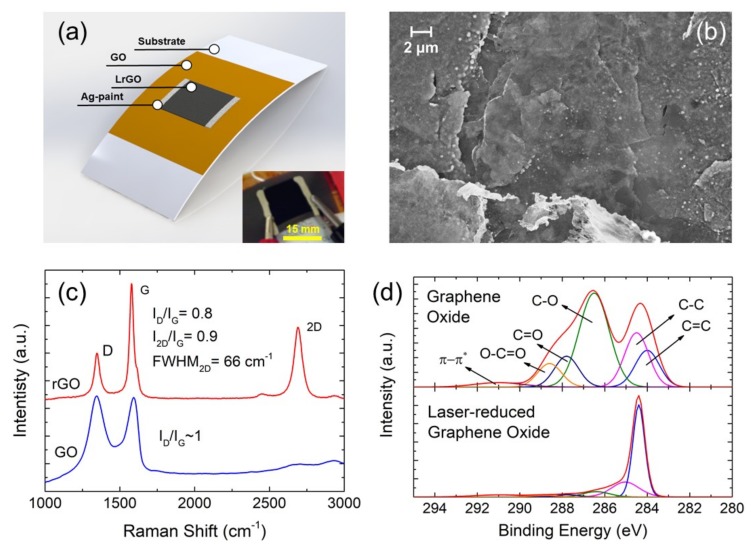
(**a**) Schematic diagram of the laser-reduced graphene-oxide (LrGO) heaters. Inset shows an actual picture of one of the LrGO heater (scale bar: 15 mm). (**b**), SEM image of a laser-reduced graphene oxide sheet [20]. (**c**) Raman spectra of both graphene oxide and laser-reduced graphene oxide. (**d**) X-ray photoelectron spectroscopy (XPS) high-resolution C 1 s peak of the graphene oxide before [20] and after the laser-reduction.

The samples were fabricated using an in-house prepared GO colloid, GOc, (4 mg/mL) following a modified version of the Hummers and Offerman method; further details on the GOc synthesis path can be found in [17]. The GOc was deposited at a concentration of ~150 µL/cm^2^ on two different outstanding thermal insulators, Kapton^®^ HN polyimide films with a thickness of 125 µm (from DuPont™, Constantine, MI, USA) and mica sheets with a thickness of ~1.5 mm (model HP5M5-1 from ZT Mica, Hubei, China). After drying the deposited GO for at least 24 h at ambient temperature (relative humidity 50%), the resulting ~50 µm layer of GO was photothermally reduced using a CO_2_ laser engraving machine (8015 Rayjet-50, Trotec Laser, S.L.U., Barcelona, Spain). The laser-assisted reduction was demonstrated as a fast and scalable approach to obtain conductive patterns of laser reduced graphene-oxide (LrGO) with different lithographic patterns avoiding the use of chemicals or masks [15,18,19]. For the fabrication of the LrGO film, a laser power of ~5 W (wavelength: 10.6 µm) was applied at an engraving speed of 300 mm/s, aiming to get low sheet resistance values (~200 Ω/sq.). The laser fluence applied on the GO was high enough to achieve a high level of reduction of the samples, ensuring the absence of resistive switching effects that appear when GO is partially reduced [20]. Furthermore, this combination of laser power and engraving speed ensured the integrity of the substrates during the photothermal reduction process. Once the surface of the GO was reduced, electrical access to the heater was achieved by means of an Ag-loaded conductive paint (from RS, Corby, UK) mask-coated on its opposed edges. A schematic representation of the resulting devices is shown in Figure 1a.

### 2.2. Structural Characterization

SEM images were recorded with an NVision 40 field-emission scanning electron microscope (from Carl Zeiss, Oberkochen, Germany) at an extraction and acceleration voltage of 5 kV. Raman spectra were acquired with a JASCO 108 NRS-5100 dispersive micro-Raman spectrometer (from JASCO, Inc., Tokio, Japan) with an excitation wavelength of λ = 532 nm. XPS measurements were performed at a base pressure of 10^−10^ Torr with an Al Kα (hν = 1486.6 eV) excitation at an operating power of 450 W using an Axis Ultra-DLD spectrometer (from Kratos Analytical Ltd., Manchester, UK).

### 2.3. Transient Electrical and Thermal Characterization

The electrical and the power measurements were performed using a software-controlled low-noise power source (B2962A from Keysight, Santa Rosa, CA USA), while the temperature of the films was recorded using the Fluke TiS75 infrared camera at a sampling rate of 9 Hz and later processed with the SmartView 4.3 software (both from Fluke Corporation, Everett, WA, USA).

## 3. Results and Discussion

### 3.1. Structural Properties

The SEM image displayed in Figure 1b corresponds to an LrGO sheet [20]. The rGO showed a platelet-like crystalline structure as a result of the partial crystalline restoration of the graphene framework, as was already reported for the LrGO [21,22] as well as for the reduction of GO by stirring in hot water [23] and by chemical methods [24]. However, the two main challenges in the reduction of graphene oxide were reducing the agglomerated layers in the crystallographic structure and increasing the C/O ratio. Hereafter, we analyzed the quality of the LrGO sheets synthesized in this work. 

On the one hand, the crystallographic quality of the samples could be analyzed with the aid of Raman spectroscopy. As can be observed in Figure 1c, before the laser treatment, the Raman spectra of the GO was composed of two broad peaks, which corresponded to the D (~1345 cm^−1^) and the G peaks (~1580 cm^−1^) typically present in graphitic materials. The ratio of both these peaks (I_D_/I_G_) was associated with the number of defects in the crystalline lattice structure of graphene, which increased as the number of defects did. Therefore, based on the Raman spectra of the GO, it was clear that its structure was mainly composed of highly defect-containing graphene flakes as a result of the hydroxyl and the epoxy groups introduced by the oxidization process [25]. 

Once the GO was laser-reduced, it could be observed how the G peak became higher and narrower than the D peak, which meant a restoration, at least partially, of the hexagonal honeycomb crystal lattice of graphene and the change of carbon atoms from sp^3^ to sp^2^ hybridization [26]. This fact was also supported by the emergence of the 2D peak at ~2700 cm^−1^, whose intensity and shape were not only correlated with the number of defects but also with the number of layers of the graphene-based structure [26]. In this case, the intensity and the full width at half maximum of the 2D band relative to the G band indicated that the LrGO obtained was comparable to the few-layered graphene obtained by chemical vapor deposition (CVD) [27].

The chemical alterations induced by the laser photothermal process were also studied by XPS. As was expected, the laser reduction led to a dramatic change in the original carbon–oxygen compounds of the GO layer [28,29]. Thus, the initial carbon atomic content of ~52% increased up to 90% after the laser reduction process, whereas the oxygen presented a decrease from ~45% before the photothermal process to ~6% after, meaning an increase from ~1 to ~15 of the atomic carbon to oxygen ratio (C/O). This demonstrated that the laser photothermal process was suitable for the production of highly reduced graphene oxide (HRG) with a C/O ratio higher than that presented by other chemical methods [30,31].

A better understanding of the nature of these changes could be obtained by means of the analysis of high-resolution C 1 s spectra before and after the laser treatment, whose results are depicted in Figure 1d. As can be seen, the laser photothermal process was able to remove most of the oxygen-containing functional groups (hydroxyl, epoxy, and carbonyl) presented as defects in the raw material and turn the hybridization of the carbon domains from sp^3^ to sp^2^. Besides, these results also indicated that the level of reduction obtained was higher than that obtained for other kinds of lasers that work at lower powers [20]. Therefore, these experiments together with the Raman results confirmed the proper reduction of the GO. 

### 3.2. Electrical and Thermal Properties

**Figure 2:**
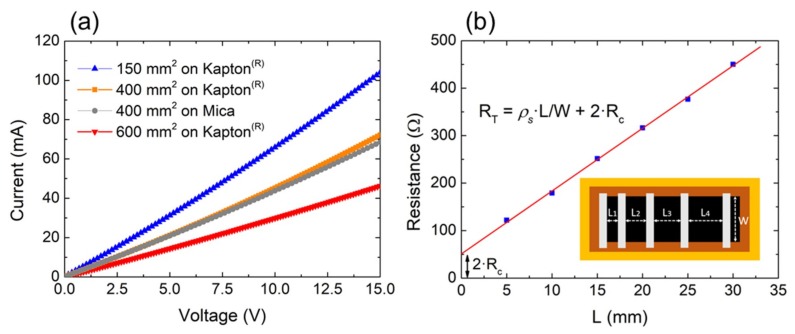
(**a**) Current–voltage (I–V) curves for different LrGO films on flexible substrates: 150 mm^2^ (L = 10 mm, W = 15 mm), 400 mm^2^ (L = 20 mm, W = 20 mm), 600 mm^2^ (L = 30 mm, W = 20 mm). (**b**) Total resistance (R_T_) as a function of the distance between consecutive contacts (L_i_) and its relationship with sheet resistance (*ρ_s_*), contact resistance (R_c_), and dimensions (L, W).

The electrical properties of the LrGO heaters were studied for different dimensions (150 mm^2^, 400 mm^2^, and 600 mm^2^) using both Kapton^®^ and mica as supporting substrates. As presented in Figure 2a, the heaters exhibited a linear relationship between voltage and current, indicating a good ohmic behavior in all cases. Note that the different slopes of the samples were associated with the different aspect ratios and the contact resistances (R_T_ = *ρ_s_* L/W + 2R_c_). Both sheet resistance (*ρ_s_*) and contact resistance (R_c_) were obtained by means of the transmission line model (TLM) [32], whose results for the 150 mm^2^ are depicted in Figure 2b. In this way, we obtained a sheet resistance of around 200 Ω/sq and a residual contact resistance (R_C_) of ~25 Ω with no dependence on the substrate, which demonstrated a proper tuning of the laser parameters. This demonstrated that an only-laser fabricated LrGO heater could achieve results comparable to those reported for Ag-doped LrGO (158.7 Ω/sq.) [10] and electrochemically exfoliated graphite (159.0 Ω/sq.) [33]. 

**Figure 3:**
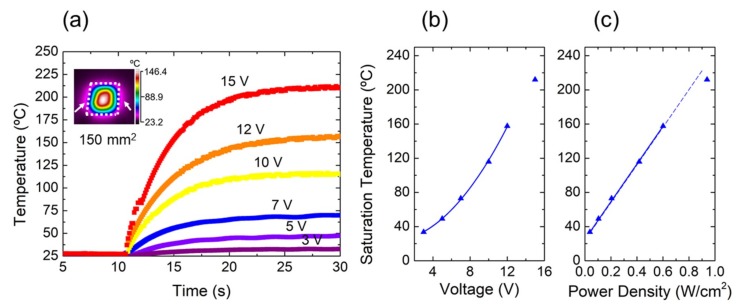
Temperature profiles of a 150 mm^2^ LrGO-based heater on Kapton^®^. (**a**) Time dependent characteristic of the heater. Inset shows a thermal image of the LrGO heater (arrows indicate the contacting sides). (**b**) Saturation temperature as a function of the voltage applied. (**c**) Saturation temperature as a function of the input power density.

The time dependence of the electrothermal characteristics of the heaters was also studied at different operation voltages in ambient conditions. For that purpose, we applied different voltage steps (up to 15 V) starting at 10 s and monitoring both current and temperature over time. The results obtained for a rectangular heater with an area of 150 mm^2^ are depicted in Figure 3. Thus, as can be observed in Figure 3a, once the step was applied, the temperature on the surface of the heater started to increase exponentially until reaching a steady-state situation. Besides, the higher the voltage applied to the heater was, the higher the final temperature was, as shown in Figure 3b. It is worth mentioning that the parabolicity of the pattern in this figure was interrupted at the higher voltage since radiative losses could be estimated in 0.032 W/cm^2^ (>5%) at this temperature according to Stefan–Boltzmann law (P/A~*σ*T^4^, with *σ* = 5.67 × 10^−8^ W/m^2^K^4^). The relationship between the steady-state temperature and the voltage applied defined the performance of the heaters in terms of transduction of electrical energy into joule heating [11]. Thus, in this case, the heaters presented steady-state temperature of ~215 °C when a voltage of 15 V was applied, demonstrating higher transduction efficiency than those reported by several works in literature, e.g., for electrochemically exfoliated graphene (139 °C at 30 V) [33], multi-walled carbon nanotubes (CNTs) (65 °C at 12 V) [34], or doped graphene (97 °C at 12 V) [9], among others. 

**Figure 4:**
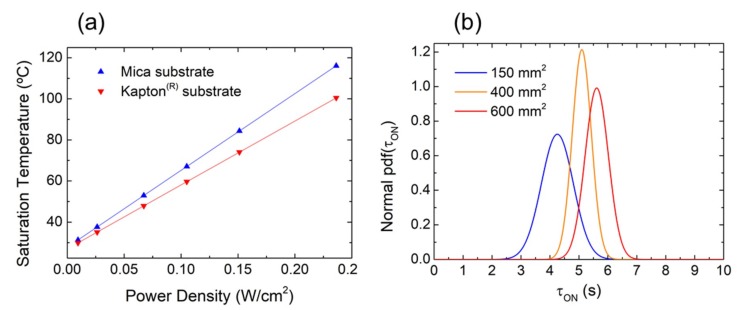
(**a**) Saturation temperature as a function of the input power density for two equal 400 mm^2^ LrGO heaters on two different substrates. (**b**) Probability density function of the response time for different heaters on Kapton^®^.

It is also interesting to study the performance of the heaters in terms of power density (W/cm^2^) since this factor has special relevance in low-power and portable applications. For that, we analyzed the saturation temperature as a function of the LrGO area of the different heaters considered in this work. In all cases, this characteristic showed an almost linear behavior, as illustrated in Figure 3c for the 150 mm^2^ heater on Kapton^®^ (with the exception of the last point of the series due to the aforementioned radiative losses) and in Figure 4a for two heaters of 400 mm^2^ on both mica and Kapton^®^ substrates. The heat transfer coefficients obtained were in the range 200–440 °C cm^2^/W, increasing with the sample area. This meant that the LrGO heaters required less power to achieve the same temperature than those reported for other materials such as laser-induced graphene (131 °C cm^2^/W) [35] or silver nanowires (134–179 °C cm^2^/W) [36,37]. It was also demonstrated that this parameter not only depended on the heat capacity of the conducting film itself but also on the substrate type, as shown in Figure 4a. This figure depicts the values of the saturation temperature as functions of the power density of two heaters with the same characteristics in terms of dimension (L = W, L × W = 400 mm^2^) and resistivity (~238 Ω) but on different substrates. It can be noted that the thermal insulation of the heaters on mica (373 °C cm^2^/W) was higher than that obtained on the Kapton^®^ substrate (311 °C cm^2^/W). This difference could be attributed to the greater roughness of the mica surface, which stunted the heat losses by means of the air gaps between its surface and the GO deposited layer [38]. 

Moreover, together with steady-state temperature, operating voltage, and power consumption, the transient thermal characteristic of the heaters is also important since many applications are subjected to strict requirements of response time, as in the case of many gas sensors [39,40]. Thus, we examined the thermal response of the heaters for different sizes and steady temperatures by means of their time constant, τ, defined as the time required by the heater to increase their temperature to 63% of the total variation [41]. Figure 4b shows the probability density function of the time constant obtained from the LrGO on Kapton^®^ heaters by applying increasing voltages up to 15 V (as shown in Figure 3a). There are many factors that influence the response time of a heater. It was demonstrated that the heating size had an effect on the time constant as well as the heat transfer coefficient (which increased with the sample area), the length to width ratio, the sheet resistance, the substrate material, and the substrate thickness [37,38,42]. Figure 4b shows how the time constant of the LrGO heaters increased from ~4.3 s to ~5.7 s when increasing the surface area. In this case, the increment on the response time could be attributed to the increase in both the size and heat transfer coefficients, as reported by Liu et al. for their CNT-based heaters [42]. In any case, this range of response time was also very promising when compared with other large-area heaters from the literature, such as the ones presented by Bobinger et al. [35] and Vertuccio et al. [43]. 

**Figure 5:**
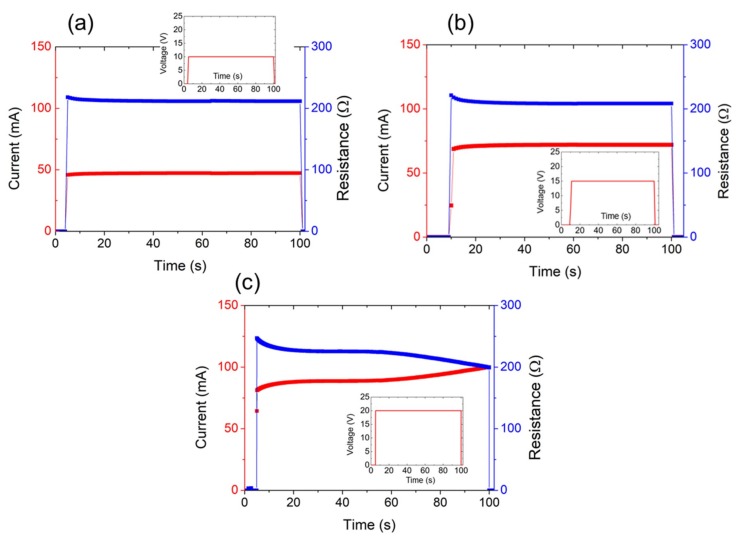
Resistance (blue) and current (red) over time of an LrGO-based heater for different step voltages of 10 V (**a**), 15 V (**b**), and 20 V (**c**).

Furthermore, when the time constant is studied, many authors consider the heater resistance to be invariable, neglecting the effect of its drift with time. However, this is not necessarily true since two effects may appear with different relevance. On one side, the heating material can present a thermistive effect responsible for relatively fast changes on the conductivity [17]; on the other side (but with more impact, especially when working at high temperatures [37,44]), the joule annealing in the LrGO film can cause a lowering of the resistance [45] through the modification of the functional groups of the GO in a similar way as the laser reduces the samples. As shown in Figure 5, whereas the heater resistance could be considered constant when fixed voltages of either 10 V or 15 V were applied between its terminals, for an input bias of 20 V, it decreased slowly (as compared with the heater time constant). This variation of the resistance influenced the transient response of the heaters, which explained the variability of the time constants when they were subjected to high thermal stress. Hence, the time constant obtained for the smallest heater, which was the one that reached the higher temperature for the lower input power, presented greater variability than the ones of the bigger samples, as previously reported by Ji et al. and Rao et al. for their silver nanowire/PEDOT: PSS film haters [37] and Au wire networks-based heaters [44], respectively. Although this fact may pose a drawback for certain applications demanding high heating needs, the power required for the appearance of joule annealing drift on the samples was estimated to be above 1.5 W, which was much higher than that normally required for these kinds of technologies and applications.

**Figure 6:**
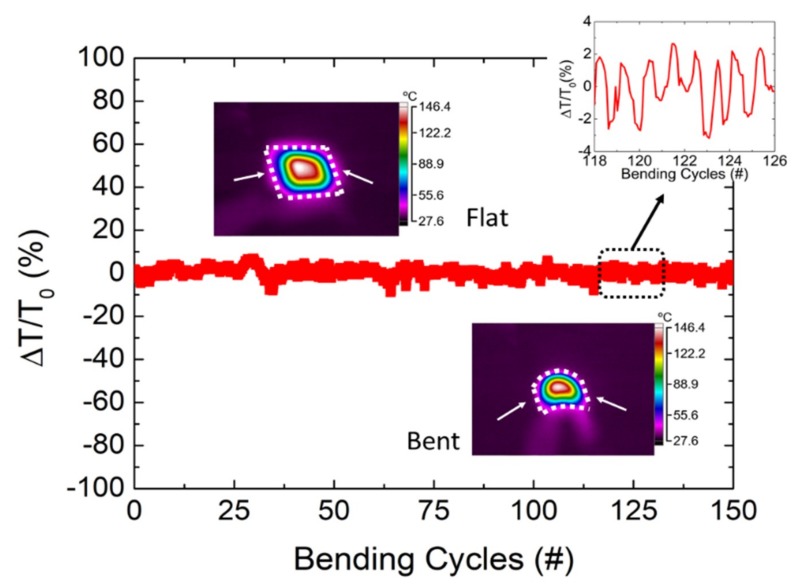
Relative change in temperature with respect to the theoretical steady-state temperature for a driving voltage of 12.5 V while bending. The heater (area: 150 mm^2^) was bent to a minimum diameter of 10 mm at a bending frequency of 0.5 Hz over 150 cycles (300 s). Insets show the infrared images of both flat and bent states (arrows indicate the contacting sides).

Further, we also analyzed the electromechanical stability of the heaters on Kapton^®^ substrate by monitoring the temperature for different conditions of bending. In this way, Figure 6 shows the relative variation of temperature [∆T/T_0_ (%)] for a 150 mm^2^ heater during 5 min (300 s) of bending with a bending radius and a frequency of 10 mm and 0.5 Hz (150 cycles in total), respectively. The driving voltage was set to 12.5 V to achieve a steady temperature of 145 °C, according to the electrothermal experiments shown in Figure 3 on a rectangular 150 mm^2^ heater. Under these conditions, the maximum changes in temperature reported were below 10 °C, resulting in a relative variation of temperature under 10%, following the periodic bending of the substrate (as shown in the inset of Figure 6). These results demonstrated good stability under bending since, under these conditions and for an interval of 150 s (even under mechanical stress), the output temperature remained consistent.

## 4. Conclusions

Laser-reduced graphene-oxide was studied as a heating element for the fabrication of flexible heaters. We presented a comprehensive study covering aspects from the production and the characterization of the raw material to the electrical and the thermal characterization of LrGO films operating as heaters for different sizes and substrates. The laser photothermal treatment was demonstrated as a fast and simple procedure to obtain conductive patterns of highly reduced graphene-oxide on flexible substrates with a competitive sheet resistance of ~200 Ω/sq. The self-heating effect of the LrGO films when voltage was applied between their terminals was studied in terms of steady-state temperature, transient response, power consumption, and size. The results illustrated that the saturation temperature depended linearly on the input power density, reporting heat transfer coefficients in the range 200–440 °C cm^2^/W. It was also demonstrated that, while the electrical conductivity did not depend on the properties of the substrate, the heat transfer coefficient did, reporting a variation of 62 °C cm^2^/W from the Kapton^®^ substrate to the mica one. The response time for the different steady-state temperatures and sizes was also analyzed, as well as its dependence on the heater area and the resistance variation. Finally, bending experiments demonstrated the stability and the consistency of the heaters when they were operating under mechanical stress. The authors believe that this technology is a big step forward in the cost-effective fabrication of high-performance heaters on large flexible substrates, which could be expanded to multiple ubiquitous applications.
